# Ultrathin Temporary Tattoo Electrodes Enable Prolonged Skin-Conformable EMG Sensing for Hip Exoskeleton Control

**DOI:** 10.3390/s26092587

**Published:** 2026-04-22

**Authors:** Michele Foggetti, Marina Galliani, Andrea Pergolini, Aliria Poliziani, Emilio Trigili, Francesco Greco, Nicola Vitiello, Laura M. Ferrari, Simona Crea

**Affiliations:** 1 The Biorobotics Institute, Scuola Superiore Sant’Anna, 56025 Pontedera, Italy; michele.foggetti@santannapisa.it (M.F.); marina.galliani@santannapisa.it (M.G.); andrea.pergolini@santannapisa.it (A.P.); aliria.poliziani@santannapisa.it (A.P.); emilio.trigili@santannapisa.it (E.T.); francesco.greco@santannapisa.it (F.G.); nicola.vitiello@santannapisa.it (N.V.); 2 Department of Excellence in Robotics & AI, Scuola Superiore Sant’Anna, 56127 Pisa, Italy; 3 Interdisciplinary Center on Sustainability and Climate, Scuola Superiore Sant’Anna, 56127 Pisa, Italy

**Keywords:** human–robot interaction, tattoo electronics, wearable devices, surface electromyography, volitional control, skin-conformal electronics, hip exoskeleton, long-term monitoring

## Abstract

**Highlights:**

**What are the main findings?**

- The tattoo electrodes maintain signal fidelity after 8 h of continuous wear and enable real-time EMG-driven assistance.
- Temporary tattoo electrodes achieve gel-grade sEMG signal quality during treadmill walking with a powered hip exoskeleton, including under the thigh cuff.

**What are the implications of the main findings?**

- Stable long-wear sEMG interfaces can simplify controller tuning by reducing the need for frequent recalibration or electrode replacement.
- Ultrathin skin-conformal electrodes can improve the usability of EMG sensing for daily wearable-robot operation.

**Abstract:**

Conventional gel electrodes are the gold standard for surface electromyography (sEMG), yet their bulkiness, stiffness, and limited gel lifetime prevents seamless day-long integration with wearable robots. We integrated ultrathin skin-conformal temporary tattoo electrodes with a powered unilateral hip exoskeleton and compared signal quality during treadmill walking against gel. In this pilot study, five healthy participants completed three consecutive walking blocks at fixed speed: (1) using gel electrodes; (2) using tattoo electrodes to compare signal quality; and (3) using the same tattoo electrodes (not repositioned) after eight hours of wear to simulate a full day of typical device use and to evaluate potential degradation in signal quality over time. Electrodes were positioned on muscles not covered by the exoskeleton interface (tibialis anterior and gastrocnemius medialis), as well as on muscles located beneath the exoskeleton cuff, which were potentially subject to motion artifacts due to the application of external forces by the exoskeleton (rectus femoris and biceps femoris, BF). Across all muscles, for both gel and tattoo electrodes, the root mean square error (RMSE) between normalized sEMG envelopes and biological activation profile was 0.069 ± 0.048, and Pearson’s correlation coefficient (*ρ*) was 0.844 ± 0.091. Re-testing the same tattoo electrode pair after eight hours confirmed day-long stability without the need for recalibration. Statistical analysis revealed no significant differences in signal quality, also when applying assistive forces, between the two electrode types and across all muscles (RMSE, all *p* ≥ 0.3125; *ρ*, all *p* ≥ 0.1250), as well as no degradation after eight hours (RMSE and *ρ*: all *p* ≥ 0.0626, uncorrected). Finally, in a proof-of-concept session, BF activity measured with tattoo electrodes was found reliable to drive hip-extension assistance in real time. Collectively, these results show that tattoo electrodes deliver signal quality comparable to gel electrodes while offering a low-profile skin-conformal interface and day-long usability, making them a promising option for enhancing EMG-based control in wearable robots.

## 1. Introduction

Control of gait-assistive exoskeletons benefits from volitional interfaces that capture the user’s intent and support seamless human–robot interaction [1]. Surface electromyography (sEMG) is the most direct non-invasive option for this purpose [2,3], and EMG-driven controllers can reduce metabolic cost and perceived effort [4]. Because next-generation exoskeletons are expected to be used daily, guiding musculoskeletal adaptation over weeks to months [5], the sensing interface must remain seamless and reliable over entire workday stretches.

In practice, sEMG electrodes often sit beneath or partly beneath exoskeleton cuffs that transmit forces and torques to the limb. With standard gel electrodes (~5 mm thick, Ø 30 mm), the added stack height under tightened cuffs can raise local pressure and shear, yielding low-frequency (<20 Hz) motion artefacts and skin irritation [6,7]. These issues are compounded by snap connectors and cable strain relief, whose rigid protrusions create hard points under the cuff and further increase pressure and shear. Over a workday, gel dehydration further increases impedance and causes amplitude drift, prompting re-gelling and replacement [8]. Commercial dry electrodes last longer but remain centimeter scale and typically cannot fit under tight cuffs [9]. Indirect muscle sensing (placing electrodes away from the interface) avoids compression but relies on task-specific synergies and gating, limiting generalizability [6].

Recently, bio-electrodes printed on flexible and soft substrates have been engineered and studied as skin-conformable dry electrodes with increased comfort for the user. Among them, “tattoo electronics” is emerging as an unconventional technology, showing outstanding performance in surface electrophysiology recordings [10]. Temporary tattoo electrodes are ultrathin skin-interfaced sensors that appear as temporary tattoos used for creative purposes. They are obtained by printing functional inks onto the commercially available and low-cost temporary tattoo transfer paper. Unlike gel electrodes, tattoo electrodes maintain stable impedance and high-quality recording capabilities under sweating, enabling all-day biopotential recording [10] while preserving a seamless skin–cuff interface under the exoskeleton.

Flexible, skin-conformable bio-electronics have begun to appear in wearable robots, including applications in soft sensor arrays on ankle–foot exoskeletons to track metabolic cost [11], stretchable microneedle patches for EMG-based control [12], and biomimetic “super-tattoos” for human–machine interfaces [13,14]. However, the direct integration of ultrathin tattoo electrodes with lower-limb exoskeletons for real-time locomotion control has not yet been documented. In addition, the durability and stability of these sensors have not been investigated during day-long use with exoskeletons in typical office settings. Notably, tattoos have been reported as a breathable interface even during sport activity (i.e., heavy sweating) [15].

In this study, we first discuss the key features of tattoo electrodes that make them suitable for integration with wearable robots and then experimentally evaluate their performance in a unilateral hip exoskeleton scenario. Our objective is to bridge a critical gap between materials science and functional rehabilitation engineering. The capabilities of tattoo electrodes were assessed through a multi-stage evaluation, ranging from benchmarking against state-of-the-art electrodes (i.e., Ag/AgCl electrodes) to prolonged day-long use. First, we examined whether tattoo electrodes can match the signal quality of Ag/AgCl electrodes, including when positioned beneath the thigh cuff of the exoskeleton. We then evaluated whether tattoo-recorded signals remain stable over time spans compatible with extended daily use and whether tattoo-based EMG signals can support a real-time exoskeleton control application. To address these objectives, we integrated tattoo electrodes into a unilateral hip exoskeleton and designed an experimental protocol comprising: (i) a baseline comparison with gel electrodes, (ii) an 8 h prolonged-wear assessment, and (iii) a proof-of-concept volitional control test.

Five healthy adults walked with a powered unilateral hip exoskeleton; we recorded from muscles under the cuff (rectus femoris, RF; biceps femoris, BF) and outside the interface (tibialis anterior, TA; gastrocnemius medialis, GM) in transparent and assistive modes. Three walking trials were executed in a fixed sequence: (1) using gel electrodes, (2) using tattoo electrodes, and (3) using the same tattoo electrodes after eight hours of wear, without replacing or repositioning the electrodes to simulate a full day of typical device use and to evaluate potential degradation in signal quality over time. To comply with recommended guidelines on electrode placement, we avoided simultaneous recording of the same muscle using both electrode types [16]. We compared the root mean square error (RMSE), Pearson’s correlation coefficient (*ρ*), and quartile coefficient of variation (CV_Q_) against a muscular activation gait baseline [17], ran statistical tests, and, in a proof-of-concept trial, used tattoo-recorded BF activity to proportionally drive hip-extension torque assistance in the exoskeleton.

Tattoo electrodes achieved an RMSE < 0.20 and *ρ* > 0.70 in all conditions. The median CV_Q_ per condition was 0.136 ± 0.034 (median 0.132 [0.116–0.145]; range 0.092–0.222). They remained unchanged after eight hours without recalibration and drove reliable volitional assistance, demonstrating fidelity comparable with the gel electrode and day-long stability.

## 2. Ultrathin Temporary Tattoo Electrodes in Electrophysiology

Fully polymeric tattoo electrodes have been successfully employed for EMG, electroencephalography (EEG), and electrocardiography (ECG) recordings. In such applications, biosignal acquisition is typically achieved using the soft organic polymer poly(3,4-ethylenedioxythiophene):polystyrene sulfonate (PEDOT:PSS) deposited on temporary tattoo paper by inkjet or screen printing [10]. When the tattoo is transferred, the conducting polymeric film comes into contact with the epidermis, enabling biosignal acquisition and transmission to the data acquisition board via flexible conducting interconnectors. Compared to gel or printed metal-based electrodes, tattoo electrodes present higher impedance due to the lower intrinsic conductivity of the conducting polymer. Impedance spectroscopy at 10 Hz confirmed |Z| ≈ 10^6^ Ω for tattoo electrodes, approximately three orders of magnitude higher than gel pads (with |Z| ≈ 2∙10^3^ Ω) pads [18]. Although higher skin–electrode impedance may reduce the sensitivity of electrophysiological recordings, considering that typical EMG amplifiers have an input impedance of 10^9^ Ω, the loading error remains below 1% of the signal, which can be considered an acceptable trade-off in performance [19,20]. In turn, PEDOT:PSS offers several advantages due to its organic nature, including intrinsic flexibility, softness, breathability, and skin biocompatibility [18]. All these properties enable high-quality recordings and superior performance compared to standard gel electrodes specifically over long use, when gel electrodes typically fail to guarantee interface stability and, consequently, signal quality [8].

Recent studies on epidermal tattoo electrodes have shown intrinsically breathable interfaces, stable dry skin coupling under perspiration, and skin compatibility over prolonged wear. These implications on skin tolerability are based on the available literature, as the present study did not include a formal dermatological assessment of skin irritation, maceration, or contact dermatitis [15,21,22].

In this study, we used PEDOT:PSS-based screen-printed tattoo electrodes to record sEMG from lower-limb muscles during walking while wearing a lower-limb exoskeleton. This study provides the first scientific evidence that this technology is durable and applicable under load conditions without degradation of the signals over time. In addition, the electrodes’ properties provide a more seamless cuff–skin interface than gel electrodes. Considering the specific conditions of use, where tattoo sensors were subjected to repeated loading through exoskeleton thigh cuffs and tattoo wires were connected to rigid leads for real-time acquisition by the EMG board, the integration of tattoo electrodes required the analysis of potential failure points, namely: (i) electrode damage, (ii) interconnect detachment or breakage, and (iii) cable-induced instability in the EMG connection. To solve these issues, we developed ad hoc electrode pads, flexible leads, and a repositionable, semi-rigid, and low-profile plug with strain relief. This assembly was customized to ensure stable recordings and reliable data collection under cuff compression.

## 3. Materials and Methods

### 3.1. Unilateral Hip Exoskeleton with Integrated sEMG Recording

**Figure 1:**
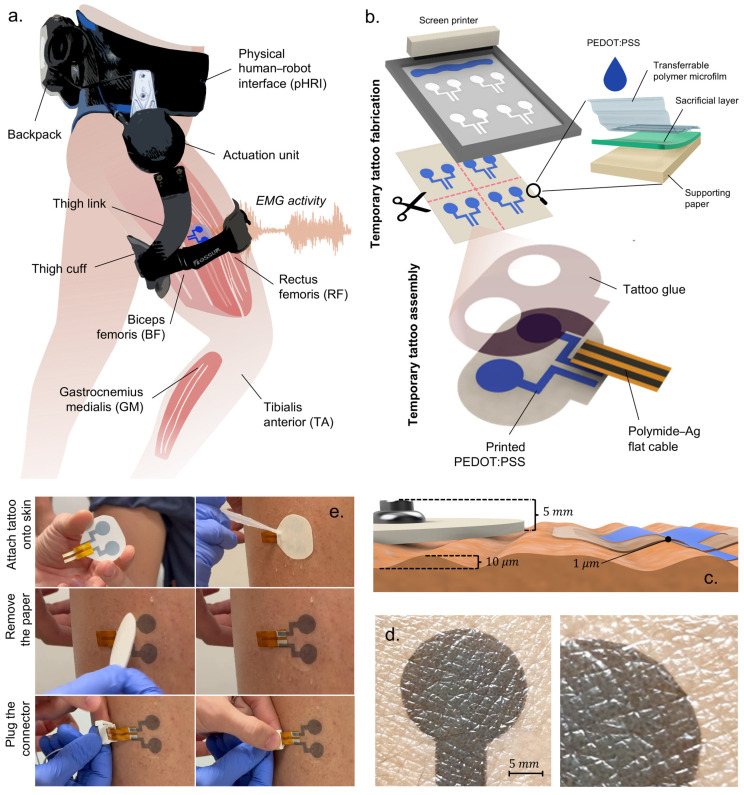
Exoskeleton and tattoo technologies overview. (**a**) Unilateral hip exoskeleton comprising an actuation unit, a physical human–robot interface (pHRI), and an electronics backpack. Surface EMG was recorded from the rectus femoris (RF) and biceps femoris (BF) beneath the thigh cuff and from the tibialis anterior (TA) and gastrocnemius medialis (GM) outside the interface. (**b**) Fabrication and assembly of the temporary tattoo electrodes. Screen-printed PEDOT:PSS on commercial layered transfer paper, oven-dried, cut, and bonded face to face to a Kapton flat cable with silver traces. (**c**) Electrode thickness comparison. (**d**) Close-up view of skin conformability, showing continuous contact and negligible edge lift; the cuff fabric can slide over the over-film, reducing shear at the skin–electrode interface. (**e**) Wet-release tattoo electrode application and connector fastening.

The unilateral hip exoskeleton used in this study was designed to supply flexion and extension torque assistance during locomotion and is shown in Figure 1a. It comprises an actuation unit positioned in line with the user’s right hip, a physical human–robot interface (pHRI), and an electronics backpack. The actuation unit has a series-elastic architecture (SEA) that delivers sagittal hip torque up to ±15 Nm at a nominal walking speed of 1.4 m/s. The SEA consists of a 70 W, 24 V brushless DC motor (Maxon Group, Sachseln, Switzerland), a 100:1 Harmonic-Drive reduction, and a planar torsional spring (240 Nm/rad). Hip angle and spring deflection are measured with 13- and 17-bit absolute magnetic encoders, namely the RMB20 and AksIM-2™ MB080 (RLS Merilna technika d.o.o., Komenda, Slovenia). Output torque measurement based on spring deflection closes a 2-pole/2-zero torque compensator running at 1 kHz [23]. The pHRI has two elements: the lower-trunk shell, which is a carbon-fiber orthosis fastened by Velcro straps and resting on the iliac crests to carry the 3.5 kg backpack, and a rigid aluminum link ending in a 70 mm wide cuff that wraps 260 mm of the mid-thigh circumference, thereby partially overlapping the rectus femoris (RF) and biceps femoris (BF) recording sites, as illustrated in Figure 1a. The cuff’s inner face is covered with a knitted air-mesh fabric (100% polyester, white, disperse dyed): the open-pore structure lets air circulate, can be disinfected between users, and minimizes shear forces at the skin–electrode interface. Strap tension is set with a winch (FIDLOCK, Hannover, Deutschland) dial-lacing system, enabling quick, repeatable tightening without adding rigid elements between the cuff and the limb. A fast clutch mounts the electronics on the left rear of the pHRI, counterbalancing the right-side actuator. Power and control are provided by a 28.8 V, 2.5 Ah Li-ion battery (Inspired Energy, LLC., FL, USA) and an sbRIO-9651 system-on-module (National Instruments, TX, USA) combining an Artix-7 FPGA and a 66 MHz ARM Cortex-A9 CPU. High-frequency tasks (1 kHz) execute on the FPGA, including SEA torque control, SPI communication with the electromyograph, and SSI communication with the motor driver.

A client router (TP-Link Systems Inc., Shenzhen, China) offers external PC connectivity. Four channels of bipolar sEMG are recorded with a Sessantaquattro Wired system (OT Bioelettronica S.r.l., Turin, Italy) from BF, RF, TA, and GM. Signals are sampled at 2 kHz, hardware bandpass filtered (10–500 Hz), rectified, and lowpass filtered at 3 Hz (2nd-order Butterworth) to obtain the linear envelope (LE). Pre-processed envelopes and raw monopolar streams are forwarded via SPI to the FPGA layer. In this study, the electromyograph was connected to two electrode types: gel and tattoo electrodes. The gel electrodes are self-adhesive ovoid pads (FIAB, Florence, Italy; 36 × 40 mm) with a 1 cm^2^ circular gel sensing area. Manufacturer specifications report a maximum impedance of 2 × 10^3^ Ω at 10 Hz and a combined offset plus internal noise of 150 µV. Overall thickness including the gel, foamed carrier, and snap connector is about 5 mm.

**Figure 2:**
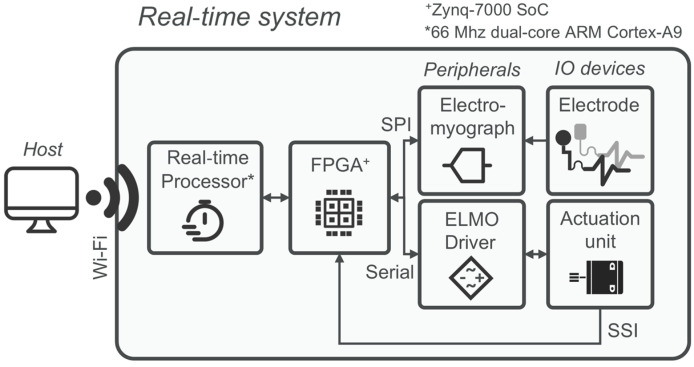
Exoskeleton hardware and real-time signal pathway. Block diagram of the system architecture: series-elastic hip actuator with encoders; electronics backpack (battery, motor driver, sbRIO-9651 with FPGA/CPU); electromyograph; and external host PC. Arrows indicate signal and communication flow among modules. The labels SPI, Serial, SSI, and Wi-Fi denote the communication interfaces used between subsystems. Surface EMG signals are forwarded via SPI to the FPGA, which also handles torque control and SSI communication with the motor driver at 1 kHz. A compact Wi-Fi router provides external PC connectivity for monitoring and logging. The asterisk (*) denotes the real-time processor, implemented as a 66 MHz dual-core ARM Cortex-A9, while the plus symbol (+) denotes the Zynq-7000 SoC.

The design, properties, and application of the tattoo electrodes used in this study are described in Section 3.3 and summarized in Figure 1b–e. Figure 2 provides a schematic overview of the device hardware architecture.

The exoskeleton can operate in three distinct control modes. In transparent mode (TM), it commands zero reference torque, ensuring the exoskeleton neither assists nor resists the user’s motion. In assistive mode (AM), it applies phase-locked Gaussian torque curves with tunable amplitude, phase, and duration. The operator independently sets the torque peaks ($P_{ext},P_{flex}$) and the duration of the assistance ($d_{ext},d_{flex}$) as a percentage of the gait phase ($\varphi_{g}$). Also, amplitudes for both flexion and extension torques are tuned through ($a_{ext},a_{flex}$). A similar control strategy can be found in [24,25,26,27].

### 3.2. Volitional Control Mode

**Figure 3:**
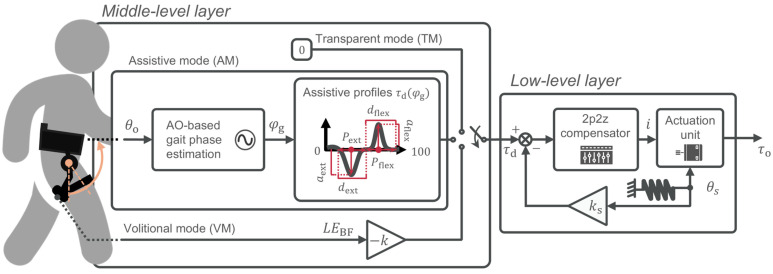
Control modes for hip assistance. Transparent mode (TM) sets zero reference torque ($\tau_{d}$ = 0). Assistive mode (AM) phase-locked Gaussian torque profiles for extension and flexion, parameterized by peak timings ($P_{ext},P_{flex}$), durations ($d_{ext},d_{flex}$) as % of gait phase ($\varphi_{g}$), and amplitudes ($a_{ext},a_{flex}$). The orange arrow indicates the input link angle ($\theta_{o}$) provided to the gait phase estimation block, while the red annotations indicate the parametrization of the desired torque profile. Volitional mode (VM) implements proportional myoelectric control, with gain k tuned so the peak equals 10% of the user’s biological hip-moment peak.

To complement the transparent (TM) and phase-locked assistive (AM) strategies, we embedded an EMG-based volitional mode (VM) in the real-time layer of the exoskeleton. In VM, the desired hip-extension torque, $\tau_{d}$, is generated directly from the linear envelope of the BF activity

(1) $$\tau_{d}=-k\cdot {LE}_{BF}$$

where ${LE}_{BF}$ is the rectified and lowpass-filtered BF signal, and the minus sign reflects the extensor action of BF, which, under the adopted convention, is represented as negative torque. The proportional gain k (units, Nm/μV) was then chosen so that the $\tau_{d}$ peak equaled 10% of the participant-specific peak of their biological hip moment, in line with the previous literature [4]. The three control modes implemented on the exoskeleton are summarized in Figure 3.

### 3.3. Tattoo Electrode Fabrication and Transfer

Tattoo electrodes for lower-limb muscle activity acquisition were fabricated by screen printing a waterborne PEDOT:PSS conductive ink (ORGACON EL-P5015; Agfa-Gevaert, Mortsel, Belgium) onto commercial tattoo paper (Silhouette America, UT, USA) using an semi-automatic printer (C920; AUREL SpA, Modigliana, Italy), following the protocol in [18]. The tattoo paper’s layered structure enables easy handling during fabrication and wet release on skin: a backing paper supports printing, while a water-soluble sacrificial layer (e.g., starch) dissolves upon wetting to laminate only the polymeric microfilm with the printed electrodes onto the epidermis. Each tattoo comprises two 1 cm^2^ pads spaced 7 mm center to center. After oven drying (60 °C, 30 min), the printed bipolar electrode is cut from the tattoo paper sheet, and the ending of PEDOT:PSS tracks is bonded face to face to a 50 µm thick polyimide (Kapton) film carrying screen-printed silver traces (obtained with ink CI-1036; Nagase ChemteX, Osaka, Japan). The Kapton film terminates in a low-profile plug that routes two flexible leads to the electromyograph.

Before tattoo transfer, the skin was cleaned using isopropyl alcohol and dried, and no conductive gel or additional abrasive skin preparation was required. Tattoos are transferred onto skin by wet release; a waterproof and breathable medical-grade polyurethane over-film (Fixomull^®^ Transparent, 37–54 µm; Essity, Stockholm, Sweden) is then laminated to improve abrasion resistance. The resulting stack is ~50 µm thick—two orders of magnitude thinner than the gel electrodes—yielding negligible protrusion under the exoskeleton cuff and allowing the cuff fabric to slide rather than shear the skin.

### 3.4. Experimental Session

This study had two objectives. First, it aimed to compare the signal fidelity of gel electrodes with that of tattoo electrodes while participants walked with the exoskeleton in TM and AM, using normalized RMSEs against physiological muscle activity profiles and correlation as comparison metrics. Second, it aimed to evaluate the durability of tattoo electrodes after prolonged use by comparing the same two metrics computed on tattoo-recorded signals at the initial time and after eight hours of continuous sensor wear on the user’s limb.

**Figure 4:**
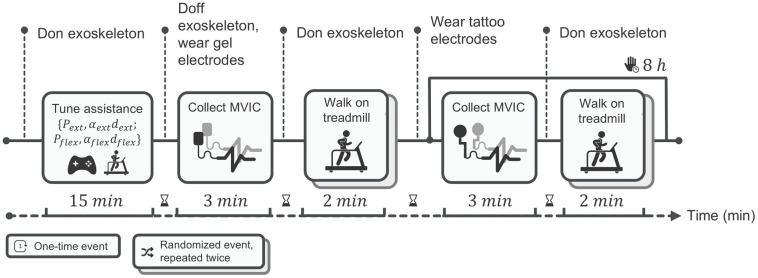
Experimental protocol and timing. After 15 min tuning/familiarization, participants completed three fixed-order conditions: Gel-T0, Tattoo-T0, and Tattoo-T8 (eight hours after with the same tattoo electrodes in place). Before each condition, participants performed MVICs for normalization. Each condition comprised two 2 min treadmill trials—transparent (TM) and assistive (AM)—in randomized order. Electrodes followed SENIAM placement; no muscle was recorded simultaneously with both electrode types. We monitored RF and BF (beneath the cuff) and TA and GM (outside). The horizontal arrow indicates the progression of time, the vertical dotted lines mark the main transition points/events of the protocol, and the stacked boxes denote randomized events repeated twice.

Five able-bodied volunteers (3 male, 2 females; 28.2 ± 1.5 yr, 177.8 ± 11.4 cm, 68.4 ± 12.1 kg) took part in the experiments. All participants provided written informed consent. Personal data were processed anonymously in compliance with the General Data Protection Regulation. The experimental procedure is presented in Figure 4.

#### 3.4.1. Assistive-Torque Running

Before data collection, each participant completed a 15 min familiarization walk with the exoskeleton, while the operator iteratively adjusted the AM Gaussian torque profiles. Peak timing for hip extension and flexion was initialized at 35% and 85% of the gait cycle, respectively, then shifted ±5% to match individual kinematics; pulse width was set to 18 ± 2% of the cycle, and peak amplitude was scaled until the measured torque reached about 10% of the participant’s biological hip-moment peak. After tuning, these parameters were kept constant throughout all subsequent trials, ensuring that any change in signal quality depended solely on the electrode type rather than on differences in exoskeleton assistance.

#### 3.4.2. Sensor Placement

Surface EMG sensors were placed on the participants’ right leg, on two muscles compressed by the thigh cuff, RF and BF, and two distal muscles that were not covered by the exoskeleton cuff, TA and GM, following SENIAM guidelines [16].

#### 3.4.3. Experimental Conditions

In accordance with recommended electrode placement guidelines, we did not record from the same muscle simultaneously with both electrode types. Tests were conducted with the following order of conditions:

- Condition 1—Gel-T0: Gel electrodes were placed, the exoskeleton donned, and walking trials in AM and TM conducted. After doffing the exoskeleton and removing the gel electrodes, the skin was cleaned with 70% isopropyl alcohol and dried.
- Condition 2—Tattoo-T0: Tattoo electrodes were applied, the exoskeleton donned, and the same walking trials repeated. After testing, participants removed the exoskeleton and engaged in normal office activities.
- Condition 3—Tattoo-T8: After eight hours with tattoo electrodes in place, the exoskeleton was re-donned and the two walking trials repeated.

Walking trials in TM and AM lasted 2 min each and were presented in random order for each condition. In total, six walking trials were executed.

Notably, because it was necessary to compare the two electrode types at baseline and to assess the durability of the tattoo electrodes without removal or repositioning, the experimental conditions were conducted in a fixed order; thus, randomization was not feasible. A potential order effect was therefore acknowledged, as it could influence the baseline comparison and lead to higher muscle fatigue during testing with the tattoo electrodes. This bias was mitigated by employing short walking trials (2 min) and incorporating appropriate rest intervals between tests. Additionally, training effects related to task order were reduced through participant familiarization with the exoskeleton testing.

### 3.5. Data Analysis

Signals were analyzed offline in MATLAB (v2025). LEs were normalized to each muscle’s maximal voluntary isometric contraction (MVIC) and time normalized over the gait cycle. For every trial, we computed the median LE profile and its interquartile range (IQR). Signal fidelity was quantified by

(2) $$RMSE=\sqrt{\frac{1}{n}\sum_{j=1}^{n}{\left({\tilde{LE}}_{j}-{\tilde{LE}}_{j}^{ref}\right)}^{2}}$$

where $\tilde{LE}$ is the trial median, and ${\tilde{LE}}^{ref}$ the normative profile from [17]. Group-level results are reported as the inter-participants’ median RMSE. Variability within each median profile was expressed with the quartile coefficient of variation

(3) $${CV}_{Q}=\frac{Q_{3}-Q_{1}}{Q_{3}+Q_{1}}.$$

To complement the RMSE, the correlation was calculated to quantify the similarity in shape between each median LE profile and the normative biological hip moment. Both signals were time normalized to the gait cycle (*n* = 101 samples); *ρ* was then obtained as

(4) $$\rho =\frac{\sum_{j=1}^{n}\left({\tilde{LE}}_{j}-\mu_{\tilde{LE}}\right)\cdot \left(M_{j}^{ref}-\mu_{M}\right)}{\sqrt{\sum_{j=1}^{n}{\left({\tilde{LE}}_{j}-\mu_{\tilde{LE}}\right)}^{2}}\cdot \sqrt{\sum_{j=1}^{n}{\left(M_{j}^{ref}-\mu_{M}\right)}^{2}}}$$

where ${\tilde{LE}}_{j}$ is the median linear envelope at sample *j*, $M_{j}^{ref}$ the reference hip-moment profile from [17], and $\mu_{\tilde{LE}}$ and $\mu_{M}$ their respective means. Values of *ρ* range from −1 (perfect anticorrelation) to +1 (perfect correlation); coefficients ≥ 0.70 were interpreted as strong similarity in accordance with conventional EMG gait analyses [28].

### 3.6. Statistical Analysis

Normality of the RMSE and correlation distributions was assessed with the Lilliefors test; given the small N=5 and violation of distributional/sphericity assumptions, non-parametric, within-participant statistics were used. For each muscle and control mode, paired Wilcoxon signed-rank tests (α=0.05, two-tailed) compared the two planned pairs: Gel-T0/Tattoo-T0 (electrode-type comparison) and Tattoo-T0/Tattoo-T8 (time-stability comparison) for both RMSE and correlation. Unless otherwise stated, *p*-values are uncorrected for multiple comparisons.

### 3.7. Tattoo-Recorded Volitional Control Proof-of-Concept Study

With the same preparation and tattoo placement, a single participant performed a proof-of-concept trial in VM. After tuning the proportional gain *k* to target 10% of the user’s biological hip-moment peak, the participant walked at 1.2 m/s for 2 min. Biceps-femoris LE, commanded torque, and SEA-measured torque were logged at 100 Hz. For each stride, the peak hip-extension torque delivered was extracted and averaged; success was defined as a mean peak within ±10% of the target without triggering safety limits. The procedure for tattoo application and a demonstration of the VM mode in operation are provided in Video S1 of the Supplementary Materials.

## 4. Results

### 4.1. sEMG Signal Fidelity with Gel and Tattoo Electrodes

**Figure 5:**
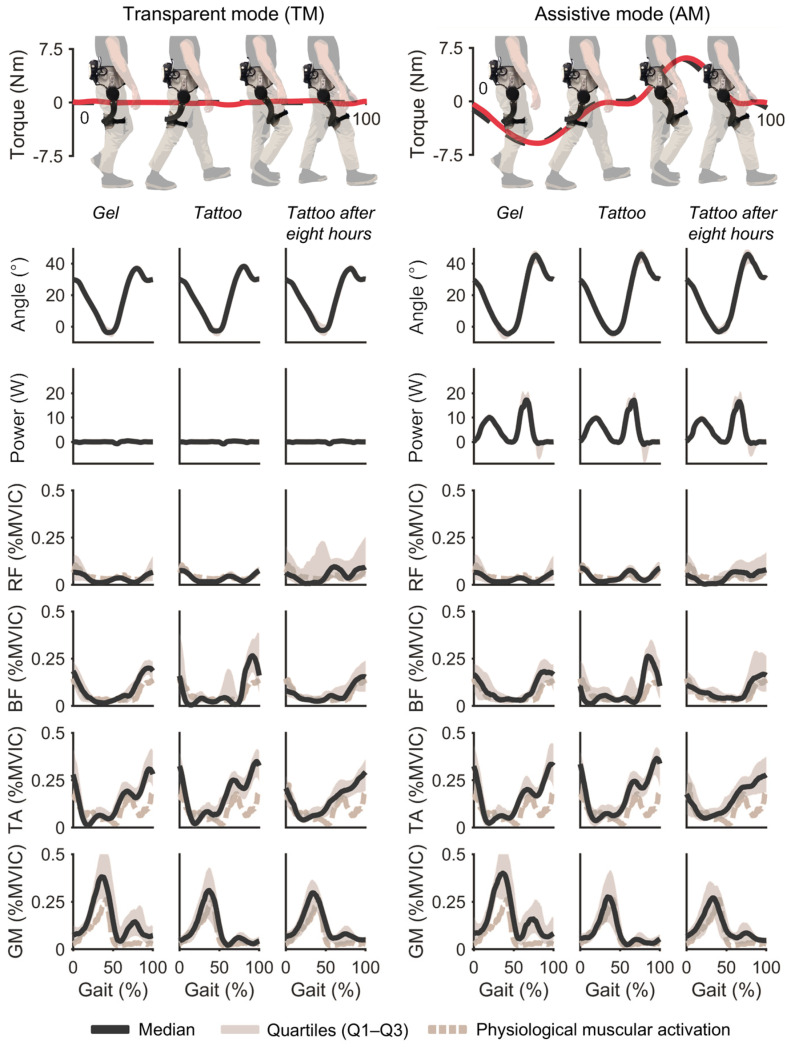
Hip joint angle, mechanical power, and linear envelopes for RF, BF, TA, and GM across conditions Gel-T0, Tattoo-T0, and Tattoo-T8 (after eight hours) for transparent (TM) and assistive modes (AM). Curves show group median with IQR shading (*N* = 5). Red lines (upper) indicate the torque delivered by the exoskeleton as a function of the estimated gait cycle.

**Figure 6:**
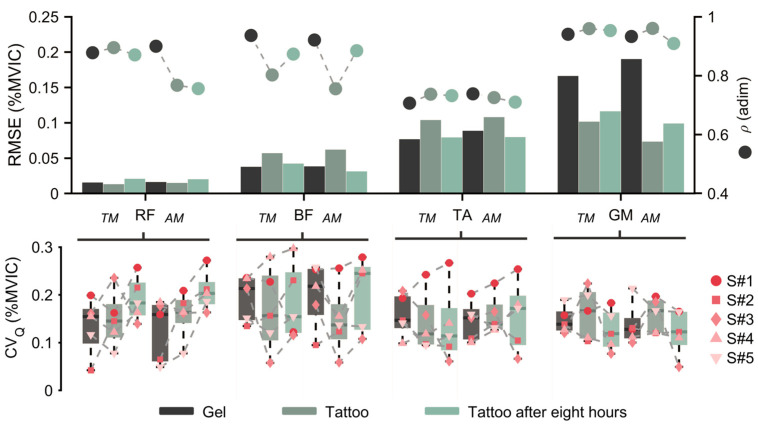
Signal fidelity and day-long metrics. (**top**) Root mean square error (RMSE, left axis) and Pearson’s correlation (*ρ*, right axis) by muscle, mode, and condition. (**bottom**) Quartile coefficient of variation (CV_Q_) distributions across participants.

Figure 5 shows the normalized LE for RF, BF, TA, and GM across the six walking trials, while Figure 6 reports the RMSE, ρ, and CV_Q_; numeric values are listed in Table 1. Across muscles and modes, the RMSE was less than 0.20 in all conditions; for BF and RF, the RMSE was less than 0.07, whereas TA and GM showed higher values consistent with their larger activation during walking. Correlation was greater than 0.70 in all conditions. The median CV_Q_ per condition (across participants) was 0.136 ± 0.034 (median 0.132 [0.116–0.145]; range 0.092–0.222). Normality (Lilliefors) and sphericity (Mauchly) tests were violated, so non-parametric paired Wilcoxon signed-rank tests were performed on RMSE, correlation, and CV_Q_.

To address the first research question, namely whether tattoo electrodes can match the signal fidelity of gel electrodes and guarantee stride-to-stride envelope consistency, including when placed directly beneath the thigh cuff, a paired Wilcoxon signed-rank comparison of Gel-T0 and Tattoo-T0 across four walking trials, all muscles, and both control modes found no significant difference in all metrics (RMSE and CV_Q_: all p≥0.3125; *ρ*: all p≥0.1250).

To answer the second research question, which was whether tattoo-recorded signals remain stable over a time span suitable for prolonged daily use as well as stride to stride, we ran paired Wilcoxon signed-rank tests comparing Tattoo-T0 and Tattoo-T8 conditions (four walking trials) for all muscles and both modes. No statistically significant effects were observed (RMSE, *ρ* and CV_Q_: all p≥0.0625).

### 4.2. Kinematics and Interaction Force

Figure 5 also reports hip-joint kinematics (median ± IQR). In transparent mode, the range of motion was 41.0 ± 5.4°, whereas in assistive mode, it was 49.8 ± 8°. The exoskeleton produced peak torques of 5.47 ± 0.94 Nm (flexion) and 5.27 ± 0.97 Nm (extension). No out-of-plane motions or abnormal peaks were observed. Mean peak mechanical power exchanged beneath the pHRI in assistive walking was 10.37 ± 2.63 W (extension) and 21.34 ± 8.13 W (flexion).

### 4.3. Proof-of-Concept Volitional Control

**Figure 7:**
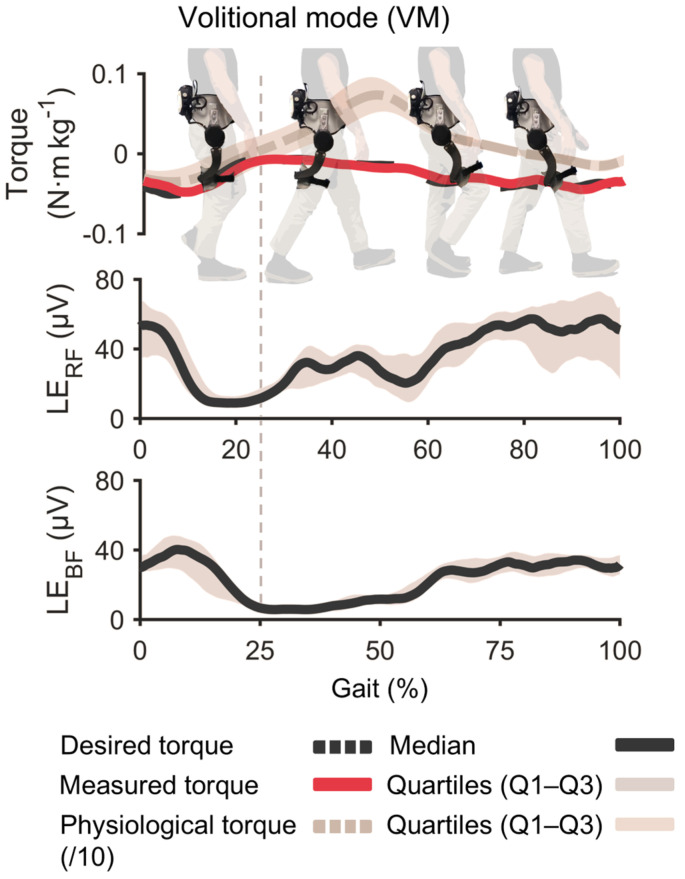
Volitional mode (VM) results. Single-participant proof-of-concept test performed after eight-hour durability block with the same tattoo electrode. Plots show biceps femoris (BF) and rectus femoris (RF) envelopes, desired torque, and measured torque over representative strides.

With the same tattoo electrode in place after the durability test, the participant completed a 2 min VM trial. The proportional gain was set to 0.07 Nm/µV to target 10% of the user’s biological hip-moment peak. Across 118 strides, the average peak assistive torque delivered was −6.80 ± 0.87 Nm, within ± 10% of the target. No saturation or safety limits were triggered, demonstrating that the tattoo-recorded BF signal can reliably drive proportional hip-extension exoskeleton assistance after a full day of wear. Figure 7 shows muscular activation LEs for the BF and RF, the desired torque, and the one delivered (measured) to the user.

## 5. Discussion

This study investigated whether ultrathin temporary tattoo electrodes can be used in hip-exoskeleton applications considering EMG fidelity and control performance. To this end, tattoo electrodes were benchmarked against gel electrodes (the current state of the art for EMG monitoring and control), and their durability was assessed during a working-day-long testing session. No statistically significant differences were observed between gel and tattoo electrodes, nor between the tattoo electrodes at the initial and final walking tests. These results have implications in wearable robotics practice, which will be discussed in detail in this section. In particular, the following three aspects will be discussed: (i) signal fidelity of electrodes placed beneath the thigh cuffs, (ii) temporal stability and failure points, and (iii) implications in volitional control design for real use.

### 5.1. Signal Fidelity Is Preserved Under the Thigh Cuff

The kinematic coupling between wearable robots and human joints is typically imperfect. Misalignments arise from biological factors (as human joints are not perfect hinges or spherical joints but combine roto-translational movements [29]) and from human–robot interfaces (as robots do not always incorporate misalignment compensation strategies along all directions [29]). Such coupling issues can cause exoskeleton cuffs to slide over the limbs, subjecting any sensory system beneath the cuffs to sliding forces in addition to forces or torques that provide movement assistance. Hence, evaluating the fidelity of sensory systems under real conditions of use with exoskeletons is paramount to determining their suitability for a specific application. In this context, the fact that tattoo electrodes are ~50 µm thick and conform to skin microgeometry allows the cuff’s fabric to slide over the electrode surface rather than shearing it. In contrast, when using gel electrodes, such sliding movements can produce motion artifacts, which are amplified by the gel’s thickness and its flow under tightening, or in the worst case, they can cause electrode detachment. This advantage of tattoo electrodes, however, comes at the cost of higher skin–electrode impedance, which was evaluated here through a direct benchmark against state-of-the-art gel electrodes and a comparison of signal fidelity.

In this study, with both electrode types and considering EMG signals of muscles beneath the exoskeleton’s cuffs (RF and BF), we observed good agreement with normative gait profiles, both in terms of signals amplitudes and correlation (RMSE < 0.07, *ρ* > 0.75), demonstrating that signal fidelity of tattoo electrodes is comparable to state-of-the-art gel electrodes.

The good performance of tattoo electrodes under the exoskeleton cuffs was confirmed by the comparison with signal quality at more distal sites (TA and GM), showing similar correlation coefficients. The higher RMSE reported in distal muscles could be attributed to the greater physiological activation during walking. Notably, the electrode contact area was the same across electrode types and muscle sites.

Although the human–robot interface was adjusted in this study to ensure proper kinematic coupling and prevent major misalignments between the user’s hip joint and the robotic joint, tattoo electrodes are expected to produce fewer movement artifacts under conditions where misalignment and shearing forces rise, such as exoskeleton migration or improper fitting [30], though this aspect was not directly demonstrated. Consistent with RMSE and correlation, the CV_Q_ remained unchanged between gel and tattoo at baseline and after eight hours.

### 5.2. Day-Long Stability: What Helps and What Can Go Wrong

Overall, when considering the temporal dimension, no systematic degradation or statistically significant differences in EMG signal fidelity (considering both RMSE and *ρ*) were observed between the tattoo baseline and the 8 h re-test.

Gel electrodes were not re-tested after 8 h, as testing the durability of both gel and tattoo electrodes over a full workday would have required at least two separate days of testing, thereby increasing the protocol complexity. In this study, we chose a single-day protocol to facilitate participant recruitment and relied on the well-established knowledge of gel electrode durability [8]. Notably, in this study, tattoo electrodes were not repositioned or recalibrated after the initial application, showing remarkable sensor stability.

Notably, the prolonged tests revealed durability issues in the entire integrated system. In one participant, an artefact caused by bending at the Kapton–wire junction was observed, highlighting interconnects as the main technical aspect requiring improvements in future experiments. Research on printed stretchable traces or magnetic snaps could reduce this weak point.

### 5.3. Implications for Controller Tuning

In the proof-of-concept trial, tattoo-recorded BF activity drove proportional hip-extension assistance in real time, achieving −6.80 ± 0.5 Nm across 118 strides; this value represents about ±10% of the biological hip torque and was achieved with direct proportional control without saturation of the sensor signals or necessity of smoothing algorithms. The lack of recalibration between morning and afternoon sessions suggests that fixed controller gains and thresholds can be carried across a typical workday when tattoos are used. Although only one single participant was tested, the result shows the feasibility of the approach.

### 5.4. Limitations and Next Steps

The main limitations of this study are the small sample size and the lack of randomization of the electrode type, as detailed in the experimental condition. Accordingly, the findings should be interpreted as preliminary evidence of the quality and durability of the tattoo electrodes. In future studies, larger and more diverse cohorts will be needed to confirm and generalize these results across wider age ranges, different anthropometries, and individuals with disabilities.

Another limitation of the study is the lack of a quantitative assessment of user comfort. Given the importance of comfort in technology acceptance, future studies will specifically investigate whether tattoo electrodes can enhance the usability of EMG systems in activities of daily living by providing more compliant and conformable interfaces, adding quantitative psychological metrics (e.g., Likert scales or Borg CR10).

Finally, the VM control experiment was performed in a single participant; the results show the feasibility of using tattoo electrodes collected under the exoskeleton cuff to drive the exoskeleton.

## 6. Conclusions

Ultrathin temporary tattoo electrodes delivered gel-grade surface EMG for hip-exoskeleton control. Across four muscles and using the exoskeleton both in transparent and assistive modes, EMG signal fidelity showed no differences between gel and tattoo electrodes at baseline, nor between baseline and the late session for tattoo electrodes, demonstrating electrode stability. Sensors placed beneath the exoskeleton cuff preserved their performance over time, with no sign of degradation due to mechanical loading. Finally, in a proof-of-concept experiment, tattoo-recorded BF activity was used to drive real-time proportional hip-extension assistance in a single participant. Together, these findings suggest that tattoo electrodes can be used as a sensing interface for low-profile, skin-conformable, and reliable EMG acquisition in wearable robotics, with potential for prolonged daily use under mechanical interaction at the human–robot interface.

## Figures and Tables

**Table 1:** Pearson’s correlation coefficient and root mean square error values for each combination.

	Transparent Mode (TM)			Assistive Mode (AM)		
Muscle	Gel	Tattoo	Tattoo After Eight Hours	Gel	Tattoo	Tattoo After Eight Hours
Rectus femoris (RF) *	0.88	0.90	0.87	0.90	0.77	0.76
	0.016	0.013	0.021	0.016	0.015	0.020
Biceps femoris (BF)	0.94	0.80	0.87	0.92	0.76	0.88
	0.038	0.057	0.042	0.038	0.062	0.031
Tibialis anterior (TA)	0.71	0.74	0.73	0.74	0.72	0.71
	0.077	0.104	0.080	0.089	0.108	0.080
Gastrocnemius medialis (GM)	0.94	0.96	0.95	0.93	0.96	0.91
	0.166	0.102	0.116	0.190	0.073	0.100

* For each muscle: the first (top) row reports the Pearson correlation coefficient (*ρ*, unitless); the second (bottom) row reports the root mean square error (RMSE, %MVIC).

## Data Availability

The original contributions presented in this study are included in the article. Further inquiries can be directed to the corresponding authors.

## Supplementary Materials

The following supporting information can be downloaded at: https://www.mdpi.com/article/10.3390/s26092587/s1, Video S1: ApplicationVideo.mov.

## Abbreviations

The following abbreviations are used in this manuscript:

AM	Assistive mode
BF	Biceps femoris
CPU	Central processing unit
CVQ	Quartile coefficient of variation
DC	Direct current
ECG	Electrocardiography
EEG	Electroencephalography
EMG	Electromyography
FPGA	Field-programmable gate array
GDPR	General Data Protection Regulation
GM	Gastrocnemius medialis
IQR	Interquartile range
LE	Linear envelope
Li-ion	Lithium ion
MVIC	Maximal voluntary isometric contraction
pHRI	Physical human–robot interface
RF	Rectus femoris
RMSE	Root mean square error
SEA	Series elastic actuator
SENIAM	Surface Electromyography for the Non-Invasive Assessment of Muscles
sEMG	Surface electromyography
SPI	Serial Peripheral Interface
SSI	Synchronous Serial Interface
TA	Tibialis anterior
TM	Transparent mode
VM	Volitional mode
